# The hemodynamic tolerability and feasibility of sustained low efficiency dialysis in the management of critically ill patients with acute kidney injury

**DOI:** 10.1186/1471-2369-11-32

**Published:** 2010-11-25

**Authors:** Heather E Fieghen, Jan O Friedrich, Karen E Burns, Rosane Nisenbaum, Neill K Adhikari, Michelle A Hladunewich, Stephen E Lapinsky, Robert M Richardson, Ron Wald

**Affiliations:** 1 Department of Medicine, University of Toronto, Toronto, ON, Canada; 2 Department of Critical Care, St. Michael's Hospital; Toronto, ON, Canada; 3 The Keenan Research Centre in the Li Ka Shing Knowledge Institute of St. Michael's Hospital; Toronto, ON, Canada; 4 Department of Critical Care, Sunnybrook Health Sciences Centre; Toronto, ON, Canada; 5 Division of Nephrology, Sunnybrook Health Sciences Centre; Toronto, ON, Canada; 6 Division of Critical Care, Mt. Sinai Hospital; Toronto, ON, Canada; 7 Division of Nephrology, University Health Network; Toronto, ON, Canada; 8 Division of Nephrology, St. Michael's Hospital; Toronto, ON, Canada

## Abstract

**Background:**

Minimization of hemodynamic instability during renal replacement therapy (RRT) in patients with acute kidney injury (AKI) is often challenging. We examined the relative hemodynamic tolerability of sustained low efficiency dialysis (SLED) and continuous renal replacement therapy (CRRT) in critically ill patients with AKI. We also compared the feasibility of SLED administration with that of CRRT and intermittent hemodialysis (IHD).

**Methods:**

This cohort study encompassed four critical care units within a single university-affiliated medical centre. 77 consecutive critically ill patients with AKI who were treated with CRRT (n = 30), SLED (n = 13) or IHD (n = 34) and completed at least two RRT sessions were included in the study. Overall, 223 RRT sessions were analyzed. Hemodynamic instability during a given session was defined as the composite of a > 20% reduction in mean arterial pressure or any escalation in pressor requirements. Treatment feasibility was evaluated based on the fraction of the prescribed therapy time that was delivered. An interrupted session was designated if < 90% of the prescribed time was administered. Generalized estimating equations were used to compare the hemodynamic tolerability of SLED vs CRRT while accounting for within-patient clustering of repeated sessions and key confounders.

**Results:**

Hemodynamic instability occurred during 22 (56.4%) SLED and 43 (50.0%) CRRT sessions (p = 0.51). In a multivariable analysis that accounted for clustering of multiple sessions within the same patient, the odds ratio for hemodynamic instability with SLED was 1.20 (95% CI 0.58-2.47), as compared to CRRT. Session interruption occurred in 16 (16.3), 30 (34.9) and 11 (28.2) of IHD, CRRT and SLED therapies, respectively.

**Conclusions:**

In critically ill patients with AKI, the administration of SLED is feasible and provides comparable hemodynamic control to CRRT.

## Background

Acute kidney injury (AKI) is a frequent complication of critical illness, and is associated with high mortality and morbidity [1]. Using contemporary definitions for AKI, renal replacement therapy (RRT) is required in 4-5% of cases [1,2]. The optimal RRT modality in these patients remains controversial.

Continuous renal replacement therapy (CRRT) has been advocated in hemodynamically unstable patients as a means of mitigating the blood pressure lability that may occur with conventional intermittent hemodialysis (IHD) [3]. However, studies directly comparing the hemodynamic tolerability of CRRT and IHD have yielded inconsistent results [4], and randomized controlled trials have not demonstrated superior survival in patients treated with CRRT [5-9]. While some studies suggest that patients treated with CRRT have a higher likelihood of renal recovery and improved renal outcomes over the long term [10,11], this has not been proven in a randomized trial. Additionally, CRRT implementation has several disadvantages, including the need for circuit anticoagulation and associated monitoring, patient immobility, intensive nursing requirements and higher overall costs [12,13,18].

Sustained low efficiency dialysis (SLED) has emerged as an alternative to CRRT in the management of hemodynamically unstable patients with AKI. SLED is administered using conventional dialysis technology used for IHD but over a prolonged period (usually 8-12 hours vs 3-4 hrs with IHD), thereby allowing for the gradual removal of fluid with less hemodynamic perturbation than IHD. Several studies have demonstrated that SLED is well tolerated in critically ill patients, with comparable ultrafiltration and solute removal to CRRT [12-16].

In this study, we examined the hemodynamic tolerability of SLED and CRRT in critically ill individuals with AKI. We also studied the overall feasibility of administering various RRT modalities in this population.

## Methods

### Population

This is a cohort study of critically ill adults who commenced RRT for AKI during admission to the medical-surgical intensive care unit (ICU), cardiovascular ICU, trauma-neurosurgical ICU or coronary care unit at St. Michaels's Hospital (Toronto, Canada) between June 2007 and July 2008. In order to exclude individuals who recovered kidney function or died shortly after RRT initiation, we did not evaluate patients who received only one RRT session. An RRT session was defined as an individual treatment with IHD or SLED or as a 24-hour period during which CRRT was prescribed. For each patient, we included up to the first three RRT sessions for these analyses. The St. Michael's Hospital Research Ethics Board approved the study and given the retrospective nature of the data collection, the need for informed consent was waived.

### Description of acute renal replacement therapy modalities

IHD and SLED were administered by hemodialysis nurses using Phoenix™ dialysis machines (Gambro, Richmond Hill, ON) and CA210 (Baxter, Deerfield, IL) and Xenium 210 (Baxter, Deerfield, IL) dialyzers. Dialysate composition and the desired ultrafiltration volume were prescribed by the treating physicians. SLED sessions were generally 8 hours in duration at a blood flow of 200 mL/min and a dialysate flow of 350 mL/min. IHD sessions were typically 3-4 hours long, with a target blood flow of 400 mL/min and a dialysate flow of 500 mL/min. CRRT, generally administered as continuous venovenous hemodiafiltration, was managed by critical care unit nurses using Prisma (Gambro, Richmond Hill, ON) and Prismaflex (Gambro, Richmond Hill, ON) machines. AN69-based filters were used for all sessions with typical blood flow rates of 100-200 mL/hour. CRRT dose was determined at the discretion of the treating physicians and the total effluent flow rate ranged between 20-35 mL/kg/hr.

### Modality assignment

RRT modality was chosen as per the clinical judgment of the consulting nephrologist with the input of the attending critical care physician. Patients who were perceived to be hemodynamically stable were treated with IHD. Hemodynamically unstable patients were typically prescribed SLED or CRRT. Hemodynamic monitoring and decisions regarding vasopressor dosing were at the discretion of the critical care team.

### Data Collection

A trained data collector compiled demographic information, reason for ICU admission, and Charlson comorbidity scores[17] on the day of RRT initiation. Bloodwork was recorded on admission to hospital, admission to ICU and on the day of RRT initiation. Severity of acute illness was described using the SOFA (Sepsis-related Organ Failure Assessment) score [18]. Systolic and diastolic blood pressure and vasopressor requirements (vasopressors included norepinephrine, phenylnephrine, vasopressin, and epinephrine) were recorded at the beginning and end of each RRT session. We also documented the nadir blood pressure during each treatment session.

### Endpoints

#### Hemodynamic instability

We defined hemodynamic instability as the composite of the following events: an intra-treatment drop in mean arterial pressure (MAP) of > 20% from the pre-treatment value or the need to escalate vasopressors. The latter was defined as the intra-treatment introduction of a vasopressor or a dose increase of a vasopressor that was already in use prior to the RRT session. Given the *a priori *expectation that patients prescribed IHD as their initial modality would be more hemodynamically stable, comparisons pertaining to hemodynamic stability focused on individuals who received SLED or CRRT. Sensitivity analyses were performed in which a MAP decline of > 10% or an absolute drop in MAP to < 70 mmHg constituted hemodynamic instability.

#### Feasibility of administration

The percentage of time during which the prescribed RRT strategy was delivered for a treatment session was the primary feasibility outcome, and was calculated as the time delivered/time prescribed × 100. The prescribed treatment time for IHD and SLED was specified by the nephrology service. By definition, CRRT was prescribed for 24 hours but if CRRT was discontinued due to evidence of renal recovery, a decision to convert to a different modality, death or withdrawal of care, then the prescribed treatment time was adjusted accordingly. We defined treatment interruption as the inability to achieve 90% of the prescribed treatment time for any given session. We categorized the primary reason for treatment interruption as follows: clotting of the extracorporeal circuit, machine malfunction, hemodynamic intolerance, nurse scheduling constraints, or patient-related. Patient-related factors included transportation for a procedure or diagnostic imaging, regardless of whether the interruption was planned prior to dialysis initiation. If a patient was switched from one modality to another, the reason for the switch was ascertained by chart review.

### Statistical analysis

Patient-level comparisons were performed after categorizing individuals by the predominant RRT modality received. This was the modality that was utilized for at least two of the three analyzed sessions. Hemodynamic and feasibility variables were evaluated during individual treatment sessions. We compared continuous variables using analysis of variance and categorical variables were compared using the Fisher exact test. We evaluated the relationship between RRT modality (SLED vs CRRT) and hemodynamic instability using generalized estimating equations, in order to account for intra-patient clustering associated with the receipt of repeated RRT sessions. Multivariable models were adjusted for age, gender, Charlson score, ICU type, SOFA score at RRT initiation, baseline estimated GFR, and vasopressor requirement prior to RRT initiation. All analyses were performed with SAS, Version 9.1.3 (SAS, Cary, NC).

## Results

**Figure 1:**
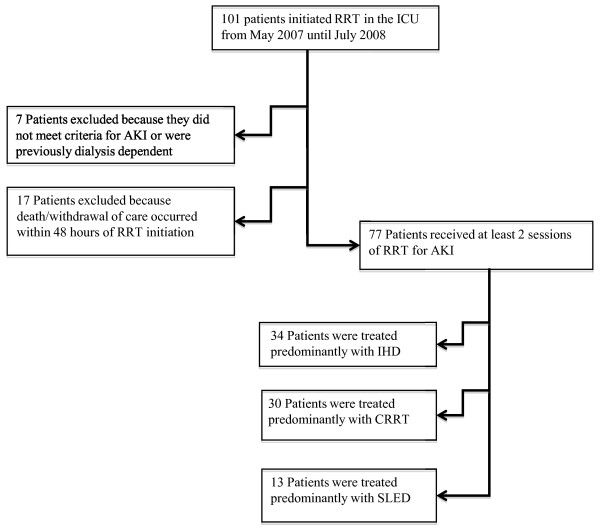
**Flow diagram of exclusion and inclusion criteria**.

During the period of observation, 101 patients received RRT in the ICU, of whom 77 patients met the eligibility criteria (Figure 1). We identified 34 patients who were predominantly treated with IHD, 30 with CRRT and 13 with SLED. Three treatment sessions were available for analysis in 69 patients and 8 patients received only 2 treatments. A total of 223 RRT sessions were analyzed.

The demographic and clinical features of the study population are summarized in Table 1. Pre-admission comorbidity was similar among patients treated with the various modalities but severity of acute illness, as reflected by the SOFA score, was substantially higher in patients who received SLED or CRRT, as compared to IHD. There was no significant difference in SOFA score at RRT initiation between patients who received SLED or CRRT (p = 0.18). Serum creatinine values at the time of RRT initiation were significantly higher in patients treated with IHD.

**Table 1:** Baseline characteristics of patients divided into groups based on the predominant renal replacement modality

		**IHD**^**a**^ (n = 34)	**CRRT**^**a**^ (n = 30)	**SLED**^**a**^ (n = 13)	P-value
**Male (%)**		20 (58.8)	25 (83.3)	8 (61.5)	0.08

**Age in years**		65.4 ± 16.4	61.5 ± 17.5	63.4 ± 10.3	0.56

**Charlson score**		2.9 ± 2.1	2.9 ± 2.5	2.5 ± 2.2	0.83

**Median serum creatinine at RRT initiation (μmol/L)**		427 (326-612)	341(259-316)	390 (352-449)	0.02

**Mean BUN at RRT initiation**		32.9 ± 18.0	25.4 ± 13.4	27.4 ± 14.1	0.16

**SOFA score at RRT initiation**		9.9 ± 3.5	15.7 ± 3.6	14.0 ± 4.1	0.0001

**ICU Type (%)**	**MSICU**	19(55.9)	24 (80.0)	7 (69.2)	0.16

	**CVICU**	11 (32.4)	3 (10.0)	2 (15.4)	

	**CCU**	4 (11.8)	2 (6.7)	1 (7.7)	

	**TNICU**	0	1 (3.3)	1 (3.3)	

Continuous variable are presented as mean ± SD except where indicated

^a ^Patients were classified according to "predominant" modality that was utilized for at least 2 of 3 sessions

SLED = sustained low efficiency dialysis

CRRT = continuous renal replacement therapy

RRT = renal replacement therapy

ICU = intensive care unit

MSICU = Medical-Surgical Intensive Care Unit

CVICU = Cardiovascular Intensive Care Unit

CCU = Coronary Care Unit

TNICU = Trauma-Neurosurgical Intensive Care Unit

All of the CRRT sessions were delivered with some form of anticoagulation (76% with regional citrate anticoagulation and 24% with unfractionated heparin). As compared to this, 95% and 75% of the SLED and IHD sessions, respectively, were delivered without anticoagulation. The remainder were performed with unfractionated heparin.

Overall, 39 (51%) patients died prior to hospital discharge. In-hospital mortality was 35%, 62% and 63% in patients treated predominantly with IHD, SLED, and CRRT, respectively.

### Hemodynamic tolerability

Pre-treatment MAP was similar for CRRT and SLED sessions (74.1 ± 10.0 vs 76.4 ± 13.1 mmHg, respectively, p = 0.34), but pressors were employed at the onset of therapy more frequently with CRRT (72% vs 49% for SLED, p < 0.01). Ultrafiltration volume was similar in both treatment groups (Table 2).

**Table 2:** Hemodynamic tolerability of CRRT vs SLED

	CRRT (n = 86)	SLED (n = 39)	p-value
MAP prior to treatment session (mmHg)	74.1 ± 10.0	76.4 ± 13.1	0.34

Vasopressor requirement prior to RRT session (%)	62 (72.1)	19 (48.7)	0.01

Volume ultrafiltered per session (mL)	1823 ± 1464	1915 ± 1302	0.74

Sessions associated with > 20% reduction in MAP (%)	16 (18.6)	15 (38.5)	0.02

Sessions with vasopressor escalation (%)^a^	34 (39.5)	10 (25.6)	0.13

Unstable sessions^b ^(%)	43 (50.0)	22 (56.4)	0.51

Continuous variables are presented as mean ± SD. Categorical variables are presented as number of sessions (%).

SLED = sustained low efficiency dialysis

CRRT = continuous renal replacement therapy

MAP = mean arterial pressure

^a^Includes any increase in pressor dosage, as well as initiation of pressors

^b^Defined as a treatment associated with a > 20% intra-treatment reduction in MAP or a treatment on which an escalation in pressor requirement occurred

SLED therapies were more frequently associated with a > 20% decline in MAP (38.5% vs 18.6% of CRRT sessions, p = 0.02). On the other hand, pressors escalation was observed more often during CRRT sessions (39.5% vs 25.6% of SLED sessions, p = 0.13). Hemodynamic instability, as defined by a composite of a MAP decline of > 20% or a need to escalate pressors, occurred in 56.4% of SLED sessions and 50.0% of CRRT sessions (p = 0.51). In a multivariable analysis that accounted for intra-patient clustering, the adjusted odds ratio for hemodynamic instability associated with SLED was 1.20 (95% CI, 0.58-2.47), as compared to CRRT.

Only one patient switched modality from SLED to CRRT due to hemodynamic instability on SLED. This patient completed one SLED treatment achieving a total treatment time of 8 hours and the prescribed ultrafiltration of 4.0 L. However, he experienced a drop in MAP of greater than 20% and was switched to CRRT for subsequent therapies. He then completed two CRRT sessions achieving an ultrafiltration of 1.3 L and 2.0 L, respectively, with no hemodynamic instability.

When the composite definition of hemodynamic instability was modified to include a more modest MAP reduction of 10%, or when hemodynamic instability was simply defined as an intra-treatment nadir systolic blood pressure of < 70 mmHg, no differences between SLED and CRRT were noted.

### Feasibility of administration

Of the prescribed treatment time, 96%, 86%, and 89%, was delivered in IHD, SLED and CRRT, respectively (Table 3). The proportion of sessions discontinued prior to the delivery of 90% of the prescribed time was 16, 35 and 28%, for IHD, SLED and CRRT, respectively. Nursing availability was the most common reason for treatment interruption in IHD. Technical issues (eg, circuit clotting) caused most CRRT interruptions. SLED sessions were most frequently interrupted due to a need to curtail treatment duration for patient transport out of the ICU. Few sessions were curtailed due to hemodynamic instability in any of the treatment modalities

**Table 3:** Prescribed treatment time delivered of each RRT treatment analyzed, and reasons for early treatment discontinuation

		All modalities	IHD (n = 98)	CRRT (n = 86)	SLED (n = 39)
Percent time delivered/time prescribed			96.1 ± 8.2	85.8 ± 22.9	89.4 ± 20.5

Mean time delivered ± SD in hours			3.0 ± 0.7	19.7 ± 6.4	6.8 ± 1.8

Sessions on which < 90% of prescribed time was delivered^a^		55 (24.9)	16 (16.3)	30 (34.9)	11 (28.2)

Reason for delivery of < 90% of prescribed treatment time	Filter clotting	10	0	9 (30.0)	1 (9.1)

	Machine-related	12	1 (6.7)	10 (33.3)	1 (9.1)

	Hemodynamic instability	3	1 (6.7)	1 (3.3)	1 (9.1)

	Patient-related	15	1 (6.7)	8 (26.7)	6 (54.6)

	Nursing constraints	16	12 (80.0)	2 (6.7)	2 (18.2)

Continuous variables are presented as mean ± SD. Categorical variables are presented as number of sessions (%).

IHD = intermittent hemodialysis

SLED = sustained low efficiency dialysis

CRRT = continuous renal replacement therapy

Fifteen patients switched RRT modality within the first 3 days of therapy and the reasons for this are summarized in Table 4.

**Table 4:** Reasons for modality switch within first three RRT sessions, based on initial modality used

Initial modality	Switched to SLED	Switched to CRRT	Switched to IHD
**SLED**	--	hemodynamic intolerance (n = 1) limited nursing availability (n = 1)	improved hemodynamics (n = 3)

**CRRT**	none	--	improved hemodynamics (n = 4)

**IHD**	hemodynamic intolerance (n = 4) deteriorated clinical status (n = 1)	hemodynamic intolerance (n = 1)	--

SLED = sustained low efficiency dialysis

CRRT = continuous renal replacement therapy

IHD = intermittent hemodialysis

## Discussion

In a cohort of critically ill patients with AKI requiring RRT, we demonstrated that the hemodynamic tolerability of SLED did not differ significantly from CRRT. In addition, SLED was feasibly accomplished as reflected by the delivery of over 85% of the prescribed treatment duration.

The theoretical attractiveness of CRRT emanates from the putative ability to remove fluid and solutes in a hemodynamically favorable manner while tailoring ultrafiltration to the patient's evolving clinical status. However, studies comparing CRRT and IHD, using variable definitions of hemodynamic tolerance, have not demonstrated a consistent hemodynamic superiority in CRRT-treated patients [4-8] (Table 5). This fact, compounded by the absence of a mortality benefit with CRRT [5-9] and the logistic demands and resource intensiveness of this modality [16,19], has impelled clinicians to seek alternate means of providing RRT to hemodynamically unstable patients with AKI. Using a clinically relevant endpoint for hemodynamic instability, our findings suggest that SLED is a viable alternative in the majority of patients who would be typical candidates for CRRT.

**Table 5:** Randomized controlled trials comparing the hemodynamic tolerability of CRRT and IHD

	CRRT (n)	IHD (n)	Definition of Hemodynamic Tolerability	Outcome
**Misset et al ^a^**[4]	27	27	Amplitude of MAP change (lowest recorded every hour to highest recorded), and episodes of MAP reduction by > 10 mmHg	No significant difference (p = 0.72 and 0.73, respectively)

**Augustine et al **[6]	40	40	Difference between mean MAP in 12 hours prior to RRT and during RRT	Significant drop in MAP during IHD (p = 0.04)

**Uehlinger et al **[7]	70	55	Number of hypotensive events (MAP < 65) during RRT	No significant difference (p = 0.36)

**Vinsonneau et al **[8]	175	184	Number of hypotensive events (SBP < 80, or drop of greater than 50 mmHg) during RRT	No significant difference (p = 0.47)

^a^A cross-over study with a 24-hour wash-out period

MAP, mean arterial pressure; SBP, systolic blood pressure

SLED has numerous practical advantages over CRRT. SLED can be performed without the need for systemic anticoagulation and recent data have suggested reduced costs as compared to CRRT [14]. The nocturnal administration of SLED, where available, allows patients to be transported outside the critical care unit during the daytime hours for routine tests and procedures, without concern about interrupting the RRT session.

We observed a higher frequency of hypotensive episodes in patients receiving SLED, and this was countered by a tendency for more frequent vasopressor escalation to manage hypotension in patients receiving CRRT. We speculate that a possible reason for this difference relates to the fact that SLED is administered by hemodialysis nurses, who have considerably more experience managing hypotension during dialysis sessions and may be more tolerant of transient hypotensive episodes, whereas CRRT is administered by ICU nurses who are more accustomed to respond to altered hemodynamics with vasopressor titration.

Our findings contribute to an expanding literature supporting the use of SLED in critically ill patients in circumstances where CRRT would typically be considered. Several studies have shown that SLED achieves adequate solute removal with acceptable hemodynamics [12-14,16]. However, hemodynamic stability has been variably defined. Some studies designated hemodynamic instability by the number of hypotensive events using absolute thresholds [12,16], while others observed changes in blood pressure before and after dialysis sessions [12,13]. Few studies, however, accounted for concurrent use and dosing of vasopressors during treatment. In clinical practice, drops in blood pressure may be tempered by the introduction or escalation of vasopressors. For this reason, we felt it was vital to combine decrements in blood pressure and changes in vasopressor dosing in order to arrive at a more comprehensive and clinically meaningful definition for hemodynamic instability.

To date, only two small trials have compared the hemodynamic tolerability of SLED and CRRT [15,20]. These studies were small, included patients with AKI regardless of duration of RRT dependence and in one study [15] patients were only followed for 24 hours. Neither study was able to demonstrate a significant difference in hemodynamic tolerability between the two modalities. We studied SLED and CRRT in a centre where both modalities were applied in critically ill patients and only in the setting of hemodynamic instability. Patients treated with SLED and CRRT had similar blood pressure and a comparable severity of chronic and acute illness at the onset of RRT. We also incorporated an analytic technique that accounted for repeated treatments within the same patient.

Our study has several limitations. Given the observational nature of this study, we cannot rule out confounding by indication. For example, patients treated with CRRT were more likely to be on pressors at RRT initiation, suggesting that they may have been less stable than those treated with SLED. Although our findings show that in general, the hemodynamic tolerability of SLED and CRRT was similar, we can not rule out that there is a subset of patients in whom CRRT is better tolerated. The choice of RRT modality was left to the discretion of the attending nephrologist and intensivist, who may have had compelling reasons for their choice which we could not capture in this retrospective analysis. Future work in this area should aim to clarify factors that inform decision-making around RRT modality. Since SLED was administered during daytime hours in our centre, SLED sessions were still interrupted for patient transportation out of the critical care unit. In addition, since this was a single centre study, our results may not be readily applicable to other settings.

We used an arbitrary cutoff of an intra-treatment MAP decline of 20% as part of the composite outcome for hemodynamic instability. Nonetheless, our results remained robust in sensitivity analyses that used different thresholds to define instability. Finally, hemodynamic instability, irrespective of definition, is a surrogate endpoint and may not be predictive of patient-relevant outcomes such as mortality and persistent dialysis dependence. However, since there is no data to favour one modality or another with respect to these hard endpoints, we believe that RRT-associated hypotension is a clinically relevant endpoint as it determines whether the therapy can be safely and practicably delivered to the patient.

## Conclusions

SLED is a well-tolerated and feasible RRT modality in the majority of critically ill patients with AKI. Within the limits of this observational study, SLED had comparable hemodynamic tolerabilty to CRRT, and was feasible to administer with a high rate of achievement of prescribed therapy duration. Larger studies will need to clarify the impact of SLED on patient survival and kidney function recovery.

## Abbreviations used in this paper

SLED: sustained low efficiency dialysis; AKI: acute kidney injury; RRT: renal replacement therapy; CRRT: continuous renal replacement therapy; IHD: intermittent hemodialysis; ICU: intensive care unit; MAP: mean arterial pressure; GFR: glomerular filtration rate; SOFA: sepsis-related organ failure assessment; RCT: randomized controlled trial.

## Competing interests

The authors declare that they have no competing interests.

## Authors' contributions

RW conceived the study, and participated in its design and coordination and performed the statistical analysis. HF conceived of the study, participated in its design, carried out the data acquisition and the statistical analysis. JF, KB, NA, MH, SL, RN and RR participated in the design of the study. RN also performed some of the advanced statistical analyses. All authors read and approved the final manuscript.

## Pre-publication history

The pre-publication history for this paper can be accessed here:

http://www.biomedcentral.com/1471-2369/11/32/prepub
